# Effect of maternal voice on sleep quality in children following cardiac surgery: protocol for a randomized controlled trial

**DOI:** 10.3389/fmed.2026.1760057

**Published:** 2026-04-07

**Authors:** Songsong Shi, Xinhong Wang, Renzhen Wang, Fengxia Du, Hao Wang, Youjia Yu, Biyu Shen

**Affiliations:** 1 Department of Cardiothoracic Surgery, Shanghai Children's Medical Center affiliated to Shanghai Jiao Tong University School of Medicine, Shanghai, China; 2 School of Nursing, Shanghai Jiao Tong University School of Medicine, Shanghai, China; 3 Department of Pediatrics, Suzhou Xiangcheng People's Hospital, Suzhou, China; 4 Department of Nursing, Suzhou Xiangcheng People's Hospital, Suzhou, China; 5 Department of Anesthesiology, Sir Run Run Shaw Hospital, Zhejiang University School of Medicine, Hangzhou, China; 6 Department of Nursing, Shanghai Children's Medical Center affiliated to Shanghai Jiao Tong University School of Medicine, Shanghai, China

**Keywords:** cardiac surgery, children, congenital heart disease, maternal voice, sleep

## Abstract

**Background:**

Children with congenital heart disease (CHD) frequently experience postoperative sleep disturbances, which can adversely affect recovery and overall postoperative outcomes. Maternal voice has been suggested as a soothing auditory stimulus with the potential to improve sleep quality in children. This study aims to evaluate the effect of auditory intervention centered on the maternal voice on sleep quality in children undergoing cardiac surgery.

**Methods:**

This prospective, randomized controlled trial will enroll 132 children with CHD undergoing open-heart surgery. Participants will be randomly assigned to either the control group or the maternal voice–based intervention group. The primary outcome is the overall sleep efficiency across the night of surgery, postoperative Day 1, and postoperative Day 2. Secondary outcomes include additional sleep parameters, pain scores, sedation scores, delirium scores, B-type natriuretic peptide (BNP) levels, cardiac troponin I (cTnI) levels, dosages of dexmedetomidine and sufentanil, cardiac intensive care unit (CICU) length of stay, total hospital length of stay, and parental satisfaction.

**Discussion:**

This trial aims to provide high-quality evidence on maternal voice to improve postoperative sleep quality in children with CHD.

**Trial registration numbers:**

Chinese Clinical Trial Registry (https://www.chictr.org.cn) ChiCTR2500111004.

## 1 Introduction

Congenital heart disease (CHD) is the most common congenital abnormality, accounting for nearly one-third of all major congenital anomalies (1). Globally, approximately 1.35 million children are born with CHD each year, representing 0.8% to 1.2% of all live births (2, 3). Nearly one in four children with CHD requires at least one surgical intervention before the age of 18 (4, 5). After cardiac surgery, children are separated from their mothers and admitted to the cardiac intensive care unit (CICU), where they are continuously exposed to nursing procedures, medical interventions, noise, light, monitoring, nutritional support, and movement within the unit (6). Additionally, postoperative factors such as the residual effects of cardiopulmonary bypass and anesthesia, cardiac medications, pain, sternotomy, drainage tubes, oxygen therapy, and restricted mobility further disrupt normal circadian rhythms and postoperative sleep quality (7). These combined stressors make children with CHD particularly vulnerable to significant postoperative sleep disturbances, which may adversely influence both immediate recovery and longer-term health outcomes.

Given the potential side effects of pharmacological interventions on sleep physiology in children, increasing attention has been directed toward non-pharmacological approaches to improving postoperative sleep. Among these approaches, the maternal voice represents a non-invasive and low-risk intervention (8). Existing evidence shows that maternal voice can improve sleep and physiological stability in newborns, especially preterm infants in neonatal intensive care units (9). This suggests that maternal voice may have similar benefits for CHD children in stressful medical environments. Maternal voice has been shown to stabilize heart rate and respiratory rate, support hemodynamic balance, and reduce blood pressure. It may also stimulate endorphin release, which can act as a natural analgesic. In addition, young children tend to have a strong attachment to their mothers. Hearing the maternal voice in an unfamiliar setting may provide emotional comfort, reduce anxiety, and promote sleep (10–13). Through these physiological and psychological pathways, maternal voice may help improve sleep quality. However, evidence is still lacking on whether maternal voice can support postoperative sleep in children undergoing congenital heart surgery.

Therefore, this randomized controlled trial will evaluate the effect of auditory intervention centered on the maternal voice on postoperative sleep in children undergoing congenital heart surgery, with the goal of generating evidence to guide clinical care.

## 2 Methods

This protocol follows the Standard Protocol Items: Recommendations for Interventional Trials (SPIRIT) guidelines, as detailed in the Supplementary material.

### 2.1 Study design and patients

**Figure 1:**
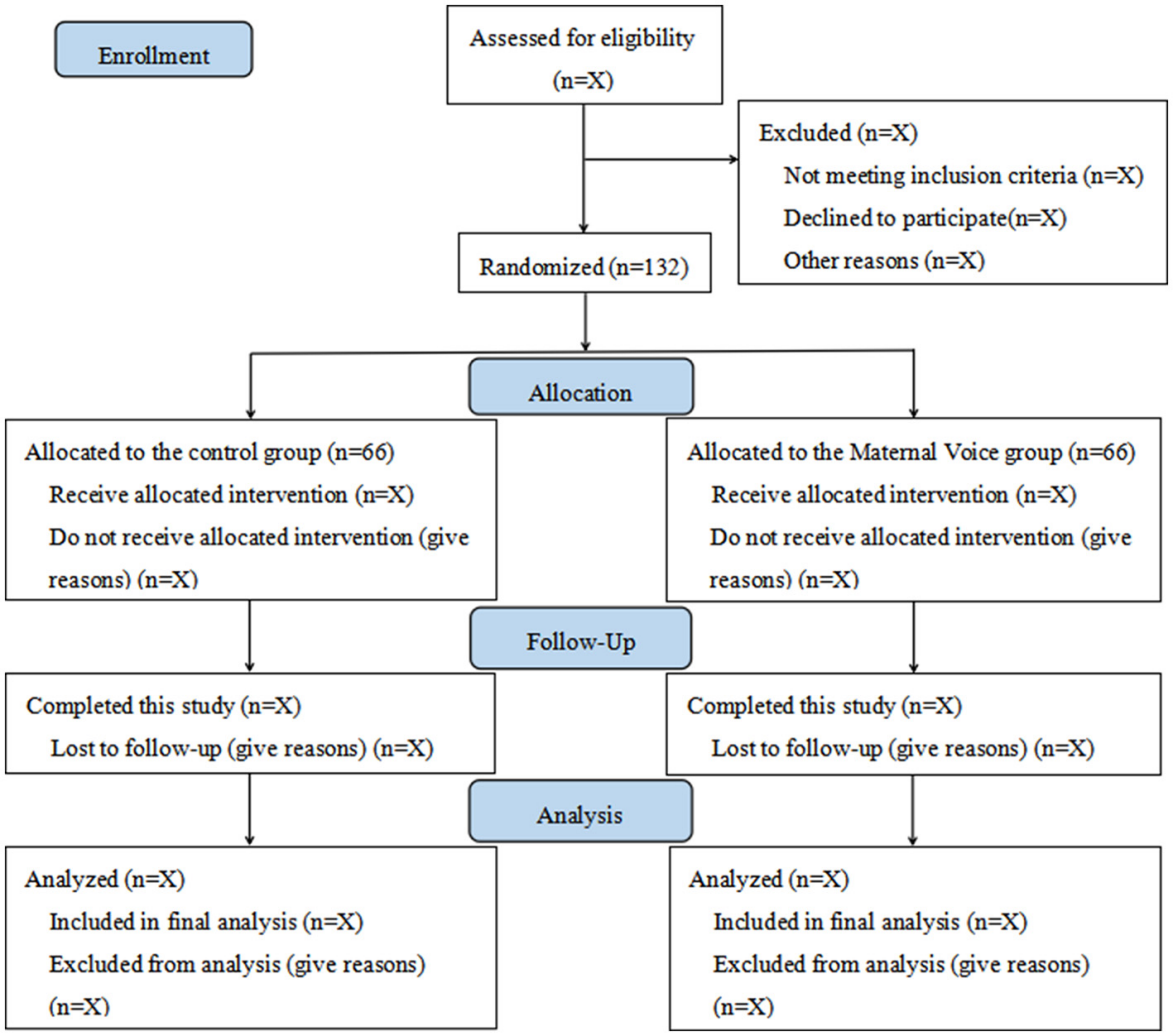
Study flow diagram.

This study is a single-center, prospective, randomized clinical trial conducted at Shanghai Children's Medical Center, affiliated with Shanghai Jiao Tong University School of Medicine. A total of 132 participants will be enrolled. Recruitment is planned from December 1, 2025, to April 30, 2026. The study flow diagram is shown in Figure 1.

### 2.2 Inclusion criteria

- Diagnosed with congenital heart disease (CHD).
- Aged between 6 months and 6 years.
- Undergoing open-heart surgery.
- Informed consent obtained from parents or legal guardians.

### 2.3 Exclusion criteria

- Require a second or subsequent surgical procedure.
- Receive extracorporeal membrane oxygenation (ECMO) therapy.
- Undergo heart transplantation.
- Have a history of epilepsy.
- Have a history of chronic pain.
- Have hearing impairment.
- Have other organ malformations or serious comorbid conditions (such as severe neurological disorders, significant respiratory disease).
- Have a postoperative endotracheal intubation duration longer than 6 h.
- Endotracheal tube could not be successfully removed before 7 p.m. on the day of surgery.

### 2.4 Primary outcome

The primary outcome of this study is the overall postoperative sleep efficiency across the first three nights after surgery. Sleep efficiency is calculated as the percentage of total sleep time divided by time in bed and will be averaged across the night of surgery, postoperative Day 1, and postoperative Day 2. Sleep will be continuously monitored from 7 p.m. to 7 a.m. using a wrist- or ankle-worn actigraphy device (Actiwatch-2, Weikang, USA), the application of activity device in children admitted to the ICU after cardiac surgery has been proven feasible (14). All actigraphy data will be preprocessed, scored, and exported using Actiware software (version 6.0.9).

### 2.5 Secondary outcomes

#### 2.5.1 Other sleep parameters

Sleep latency, total sleep time, wake time during sleep, and the number of nighttime awakenings will be collected using actigraphy. Actigraphy-derived sleep parameters have been shown to provide estimates comparable to those obtained from polysomnography. The Actiwatch device contains a calibrated accelerometer and an internal memory system housed in a wristwatch-shaped casing. The accelerometer records movement at a sampling rate of 32 times per second.

#### 2.5.2 Pain scores

Pain will be assessed using the Face, Legs, Activity, Cry, Consolability (FLACC) scale developed by Merkel. The scale includes five items, each scored from 0 to 2, for a total score of 10. Higher scores indicate more severe pain. The scale has shown good reliability and validity for pain detection in Chinese children after cardiac surgery (15). Pain assessments will be recorded by the research nurse every 4 h postoperatively, the set observational points were 1 a.m., 5 a.m., 9 a.m., 1 p.m., 5 p.m. and 9 p.m. The target FLACC score range is 0–3. When the score is ≥4, a single booster dose (0.02 to 0.025 μg/kg) should be administered through the analgesia pump. An additional pain assessment should be conducted within 30–60 min after implementing pain relief measures.

#### 2.5.3 Sedation scores

Sedation and agitation will be assessed using the Richmond Agitation and Sedation Scale (RASS), which includes 10 levels ranging from +4 to−5, reflecting states from “aggressive” to “unresponsive”. Each level corresponds to a specific degree of alertness and can be evaluated through observation, verbal interaction, and physical stimulation. The RASS was a reliable and effective scale to evaluate critically ill children's sedation effect (16). RASS will be evaluated by the research nurse every 4 h postoperatively. every 4 h postoperatively, the set observational points were 1 a.m., 5 a.m., 9 a.m., 1 p.m., 5 p.m. and 9 p.m. The target RASS score range is−2 to 1 (if ≤ -3, gradually reduce the dexmedetomidine dose until discontinuation; if ≥2 points, gradually increase the dexmedetomidine dose up to 0.75 μg/kg/h). An additional sedation assessment should be performed within 30 min after dose adjustment.

#### 2.5.4 Delirium scores

Delirium will be assessed using the Cornell Assessment of Pediatric Delirium (CAPD) scale, which includes 8 items rated on a 5-point scale: “Never, Rarely, Sometimes, Often, Always.” The first four items are scored from 4 to 0, and the last four from 0 to 4. The CAPD is valid and reliable pediatric delirium screening tool (17). A total score greater than 9 indicates delirium. The scale must be applied after RASS assessment and cannot be used when the RASS score is −4 or −5. Delirium assessments will be performed by the research nurse every 12 h postoperatively, the set observational points were 9 a.m. and 9 p.m. The target CAPD score range is 0–6 points. When the score is 7–9 points, non-pharmacological interventions are recommended for prevention (e.g., early mobilization). When the score is ≥10 points, gradually increase the dexmedetomidine dose up to 0.75 μg/kg/h.

#### 2.5.5 Analgesic and sedative agents' administration

The nurse will adjust the doses of dexmedetomidine and sufentanil based on the child's sedation and analgesia scores. On the day of surgery, postoperative Day 1, and postoperative Day 2, the consumption of dexmedetomidine and sufentanil will be recorded. Sufentanil is a routinely used opioid for postoperative, a booster dose (0.02 to 0.025 μg/kg) will be administered when the FLACC score is ≥4.

#### 2.5.6 CICU length of stay, total hospital stay, and parental satisfaction

Length of stay in the cardiac intensive care unit and the total length of hospitalization will be recorded at discharge. Parental satisfaction reflects the entire hospitalization experience, will be assessed using a 0 to 100 mm visual analog scale (VAS) on the day of discharge, with 0 indicating “very dissatisfied” and 100 indicating “most satisfied imaginable.”

### 2.6 Exploratory outcomes

#### 2.6.1 Laboratory examination

B-type natriuretic peptide (BNP) levels will be measured to assess cardiac function, and cardiac troponin I (cTnI) levels will be measured to evaluate myocardial injury. Laboratory tests will be performed every 12 h postoperatively by nurse, the collection time point were 9 a.m. and 9 p.m. Meanwhile, postoperative dataset (such as Ejection Fractions, Serum Creatinine, urine output, fluid balance, and the dose of diuretics used) will be recorded by nurse.

### 2.7 Adverse events

If an adverse event occurs during the intervention period, detailed documentation must be recorded in the original medical record, including the time of occurrence, clinical manifestations, treatment provided, duration, outcome, and the event's potential relationship to the trial. In the event of a serious adverse event, a serious adverse event report form must be completed and submitted to the clinical research center within 24 h.

### 2.8 Randomization and blinding

An independent researcher will generate the randomization sequence using an online randomization tool (https://www.sealedenvelope.com/simple-randomiser/v1/lists) with a 1:1 allocation ratio. The randomization results will be placed in sealed, opaque, sequentially numbered envelopes. Two hour before surgery, a research nurse will open the next envelope in sequence and assign each child to either the maternal voice–based intervention group or the control group.

Due to the nature of the maternal voice–based intervention, postoperative care providers cannot be blinded to group allocation. Children, as participants, will also not be blinded. However, parents will remain blinded, as all parents will record maternal voice clips before enrollment and will not know whether the recordings are used during the intervention. None of the non-blinded personnel or participants will be involved in participant recruitment, outcome assessment, data collection, or statistical analysis. The research nurse responsible for recruitment, the surgeons, the outcome assessors, and the statistician conducting the data analysis will remain blinded to group allocation throughout the study.

### 2.9 Study interventions

#### 2.9.1 Control group

Children in the control group will receive routine CICU care, including basic nursing care, positioning management, nutritional support, and medication therapy. No additional auditory intervention will be provided.

#### 2.9.2 Maternal voice group

Children in the maternal voice group will receive auditory intervention centered on the maternal voice in addition to routine CICU care. Before surgery, mothers will record a 5-min soothing audio clip using a smartphone or voice recorder under the guidance of the research team. The content may include comforting phrases, lullabies, or familiar melodies tailored to the child's preferences. Mothers will be instructed to call the child's name and use a calm, gentle tone. All recordings will be processed using digital audio editing software to remove background noise and then saved under the child's hospital identification number.

After surgery, children will be transferred to the CICU and will undergo routine monitoring until extubation after regaining consciousness. Maternal voice playback will be provided for 20 min before sleep on the night of surgery, the first postoperative night, and the second postoperative night, following each child's habitual circadian sleep schedule at home. If a child awakens during sleep, an additional 20-min playback session will be administered.

The speaker will be positioned 15 to 20 centimeters from the child's ear to ensure appropriate auditory stimulation. To protect hearing, the sound intensity will be monitored individually using a sound level meter placed near the speaker, ensuring a volume of ≤ 50 dB according to recommendations from the American Academy of Pediatrics (AAP, 1997). To prevent infection, the surface of the smartphones or voice recorders used for playback will be disinfected with 75% alcohol before each session.

### 2.10 Data collection and monitoring

All study data (Table 1) will be systematically recorded using standardized case report forms (CRFs). Baseline (preoperative) data will include demographic information (age, sex, height, weight, nutritional status, sleep timing/quality), type of surgery, and any comorbid conditions. We assessed the parents of the children using a 0 to 100 millimeter Visual Analog Scale (VAS) to evaluate their children's sleep quality before hospitalization and inquired about sleep timing. Intraoperative data, recorded by the surgical team, will include the duration of surgery, cardiopulmonary bypass time, and incision site. Postoperative sleep parameters will be collected and interpreted by trained sleep specialists using an actigraphy device after extubation. Duration of mechanical ventilation and additional postoperative outcomes, including pain scores, sedation scores, delirium scores, BNP levels, cTnI levels, post-extubation respiratory support, and the dosages of dexmedetomidine (maintenance dose 0.25 to 0.75 μg/kg/h) and sufentanil (maintenance dose 0.04 to 0.05 μg/kg/h; booster dose 0.02 to 0.025 μg/kg), will be recorded by trained outcome assessors. CICU length of stay and total hospital stay will be retrieved from electronic medical records, and parental satisfaction will be assessed at discharge.

**Table 1:** Schedule of patient enrolment, study interventions and outcome assessment.

Time point	Study period					
	**Enrolment**	**Allocation**	**Post–allocation**			**Close–out**
	**Pre–op visit**	**2 h before surgery**	**Night after surgery**	**Post–op day1**	**Post–op day2**	**Discharged**
Patient enrolment						
Eligibility criteria	×					
Written informed consent	×					
Demographic data	×					
Baseline characteristics	×					
Randomization/allocation		×				
Study interventions						
Maternal voice			×	×	×	
Routine CICU care			×	×	×	
Outcome assessment						
Sleep efficiency			×	×	×	
Sleep latency			×	×	×	
Total sleep time			×	×	×	
Wake time during sleep			×	×	×	
Number of wakes during sleep			×	×	×	
Pain scores			×	×	×	
Sedation scores			×	×	×	
Delirium scores			×	×	×	
BNP value			×	×	×	
cTnI value			×	×	×	
Dose of dexmedetomidine			×	×	×	
Dose of sufentanil			×	×	×	
CICU length of stay						×
Total hospital stay						×
Parental satisfaction score						×

According to SPIRIT statement of defining standard protocol items for clinical trials.

BNP, Brain Natriuretic Peptide; cTnI, Cardiac troponin I; CICU, Cardiac Intensive Care Unit; Pre-op, Preoperative; op-day, day of operative.

Post-op, Postoperative; SPIRIT, Standard Protocol Items: Recommendations for Interventional Trials.

All study data will be entered into a secure, password-protected electronic database under the supervision of the principal investigator. Data accuracy and completeness will be reviewed regularly. Once data entry is finalized, the database will be locked, and de-identified datasets will be transferred to an independent statistician for analysis in accordance with the predefined statistical analysis plan. All serious adverse events will be documented in detail and managed according to standard clinical protocols. The principal investigator will be notified promptly, and the Data Monitoring Committee (DMC) will conduct an independent review to assess the event's impact on participant safety and trial continuation.

### 2.11 Sample size calculation

The sample size was estimated using the G^*^Power software (version 3.1.9.7). Based on our pilot study, which included 16 children (8 in each group), preliminary estimates of sleep efficiency were obtained. The mean (M) and standard deviation (SD) of sleep efficiency were M1 = 0.772 and SD1 = 0.102 in the maternal voice group, and M2 = 0.716 and SD2 = 0.113 in the control group. Using these values, the calculated effect size was 0.52. With a statistical power of 0.80, a two-sided alpha level of 0.05, and an anticipated attrition rate of 10%, the required total sample size for this study was determined to be 132 children.

### 2.12 Statistical analysis

The Shapiro–Wilk test will be used to assess the normality of continuous variables. Normally distributed data will be presented as mean (standard deviation), whereas non-normally distributed data will be reported as median (interquartile range). Categorical variables will be summarized as numbers (percentages).

The primary outcome is the overall postoperative sleep efficiency across the first three nights after surgery. It will be analyzed using a linear mixed-effects model with fixed effects for treatment group, time, and their interaction, and a patient-level random intercept to account for repeated measurements. The primary treatment effect will be defined as the overall effect of the intervention on sleep efficiency across the three postoperative nights as estimated from this model. The model will be adjusted for age (≥6 months to < 12 months and ≥1years to ≤ 6 years), sex, type of surgery (STAT mortality category), cardiopulmonary bypass time, preoperative sleep timing/quality, and post-extubation respiratory support [high flow nasal cannula (HFNC), non-invasive ventilation (NIV), and conventional oxygen treatment (COT)], which are considered clinically relevant prognostic factors. Additionally, interactions between the treatment group and key baseline characteristics (age and cardiopulmonary bypass time) will also be examined, and interaction terms with *P*-values < 0.10 will be retained in the final adjusted model. Adjusted mean differences with 95% confidence intervals (Cis) will be reported.

Secondary continuous outcomes, including sleep latency, total sleep time, wake time during sleep, number of nighttime awakenings, pain scores, sedation scores, and delirium scores, will be analyzed using linear mixed-effects models or generalized estimating equations, as appropriate. Categorical outcomes will be analyzed using the chi-square test or Fisher's exact test. Other postoperative outcomes (CICU length of stay, total hospital stay, dosages of dexmedetomidine and sufentanil, and parental satisfaction) will be compared using independent samples *I*-tests or Mann–Whitney U tests based on data distribution.

If children are transferred out of the CICU on the third postoperative night, sleep parameters from that night will be excluded from the primary analysis but retained for prespecified sensitivity analyses.

To control for multiple testing among secondary outcomes, the Benjamini–Hochberg procedure will be applied with statistical significance set at *q* < 0.05. For all other analyses, a two-sided *P* < 0.05 will be considered statistically significant.

All analyses will follow a modified intention-to-treat (mITT) principle, including all randomized participants with at least one available post-intervention sleep measurement. Additionally, a per-protocol (PP) analysis will be conducted as a sensitivity analysis to assess the robustness of the primary findings. Missing data will not be imputed, and no interim analyses are planned. Analyses will be performed using SPSS software (version 25.0; IBM Corp., Armonk, NY).

## 3 Discussion

This randomized clinical trial will be conducted in the cardiac intensive care unit (CICU) of a tertiary children's hospital in Shanghai, China, a high-volume center specializing in pediatric cardiac surgery. The study aims to evaluate the effect of auditory intervention centered on the maternal voice on postoperative sleep in children with congenital heart disease. Sleep efficiency is the primary objective to examine its impact on key sleep outcomes. Secondary objectives include assessing additional sleep parameters and clinical outcomes such as sleep latency, total sleep time, wake time during sleep, and the number of nighttime awakenings, pain, sedation, and delirium scores, BNP and cTnI levels, dosages of dexmedetomidine and sufentanil, CICU length of stay, total hospital stay, and parental satisfaction. The trial has been designed in accordance with the Standard Protocol Items: Recommendations for Interventional Trials (SPIRIT) guidelines.

Circadian rhythm disturbances are common after cardiac surgery in children, disrupting sleep–wake homeostasis and resulting in frequent nighttime awakenings as well as impaired thermoregulation, inflammatory control, and immune stability (18). These physiological disruptions may further contribute to a higher risk of postoperative delirium, increased healthcare costs, prolonged hospitalization, and elevated mortality (19). Although medications are often used to manage pediatric sleep problems, chloral hydrate is the only FDA-approved agent, and other sedatives are used off-label with limited supporting evidence and uncertain long-term safety (20). In addition, concerns among parents and clinicians about chloral hydrate's potential for prolonged sedation, unstable breathing, and unpredictable behavioral reactions have further limited its acceptability in routine care (21).

In contrast to the limited evidence supporting pharmacological approaches, a growing body of research suggests that non-pharmacological interventions can improve sleep in hospitalized children. Reported strategies include kangaroo care, gentle touch therapy, noise reduction, music therapy, lighting adjustments, and structured nursing interventions (22). However, the feasibility of these interventions varies across clinical settings, and some may be challenging to implement consistently due to resource demands and workflow considerations. Maternal voice, by comparison, is a non-invasive, low-cost intervention that requires minimal staff involvement and aligns closely with attachment theory, which emphasizes the strong emotional bond between children and their primary caregivers. When children are separated from their mothers and transferred to the CICU, the mother's voice may serve as a surrogate attachment cue, providing comfort and a sense of security during a highly stressful and unfamiliar environment (23).

A growing body of research across neonatal and pediatric settings has demonstrated that maternal voice provides meaningful physiological and psychological benefits for hospitalized children. Studies in neonatal intensive care units have shown improvements in sleep regulation and reductions in procedural pain (24–27), while investigations in pediatric intensive care units have reported stabilization of vital signs and enhanced hemodynamic comfort (28, 29). In perioperative and diagnostic settings, maternal voice has been associated with reduced anxiety, lower postoperative pain, and decreased sedative requirements (30, 31). More recently, findings in children with congenital heart disease suggest that maternal voice may also help reduce postoperative delirium following cardiac catheterization (32). Although existing studies suggest the therapeutic value of maternal voice, its effect on postoperative sleep in children undergoing cardiac surgery has not been systematically examined. Given the high prevalence of sleep disruption after cardiac surgery and its clinical consequences, a safe, non-invasive, and feasible intervention is urgently needed. This trial seeks to fill this gap by evaluating whether auditory intervention centered on the maternal voice can improve postoperative sleep in children with congenital heart disease.

This study has several limitations. First, while actigraphy is practical and widely used in pediatric sleep research, it cannot capture sleep architecture in the same way as polysomnography (PSG), which may result in a less comprehensive assessment of sleep in this study. Second, the primary outcomes were assessed only during the first three postoperative nights, and no long-term follow-up was conducted, which limits our ability to evaluate the sustained effects of the intervention. Third, this was a single-center study conducted in a CICU setting, which may limit the external validity of the findings and their broader applicability to other pediatric cardiac populations. Fourth, Children requiring prolonged postoperative mechanical ventilation were excluded because actigraphy monitoring was initiated after extubation to ensure data completeness on the night of surgery, which may limit generalizability to patients with more complex postoperative courses. Finally, the observed effects may reflect the combined influence of multiple factors–including auditory stimulation, structured intervention exposure, and attention-related effects. Future studies incorporating attention-matched control groups and unfamiliar voice comparators will be needed to disentangle these components.

In summary, this randomized controlled trial is designed to evaluate the effect of auditory intervention centered on the maternal voice on postoperative sleep in children with congenital heart disease. As a non-invasive, low-cost, and low-risk non-pharmacological approach, auditory intervention centered on the maternal voice may offer a practical and effective strategy to support postoperative sleep management in this population.
